# Viral Genome Sequencing Proves Nosocomial Transmission of Fatal Varicella

**DOI:** 10.1093/infdis/jiw398

**Published:** 2016-08-28

**Authors:** Daniel P. Depledge, Julianne Brown, Jasna Macanovic, Gill Underhill, Judith Breuer

**Affiliations:** 1 Division of Infection and Immunity, University College London; 2 Great Ormond Street Hospital VZV Reference Laboratory, Great Ormond Street Hospital, London; 3 Wessex Renal and Transplant Services; 4 Departments of Clinical Microbiology, Pathology Centre, Queen Alexandra Hospital, Cosham, Portsmouth, United Kingdom

**Keywords:** fatal varicella, nosocomial transmission, whole genome sequencing

## Abstract

We report the first use of whole viral genome sequencing to identify nosocomial transmission of varicella-zoster virus with fatal outcome. The index case patient, nursed in source isolation, developed disseminated zoster with rash present for 1 day before being transferred to the intensive care unit (ICU). Two patients who had received renal transplants while inpatients in an adjacent ward developed chickenpox and 1 died; neither patient had direct contact with the index patient.

Varicella-zoster virus (VZV) causes both varicella and, after latency in sensory ganglia, herpes zoster. Universal vaccination of children has reduced the incidence of varicella and its complications in the United States, and vaccination against zoster is available for persons aged >60 years in the United States or >70 years in the United Kingdom. Neither vaccine is licensed for use in immunocompromised patients, for whom both chickenpox and herpes zoster are potentially fatal. Because transmission is thought to arise mainly through aerosolized virus from skin lesions, current advice in hospital settings is to isolate patients with diagnoses of chickenpox or widespread shingles to prevent nosocomial spread to susceptible contacts [1]. Viral genotyping provides a potential tool for linking cases where transmission is not certain [2–4]. However, where viruses are of the same clade [5], current genotyping methods cannot distinguish directly transmitted viruses from those that are unrelated or freely circulating [2]. Here we report the use of a novel method for sequencing whole viral genomes directly from residual diagnostic samples to confirm nosocomial transmission of fatal varicella in a renal transplant recipient.

## METHODS

### Patient 1

**Figure 1.**
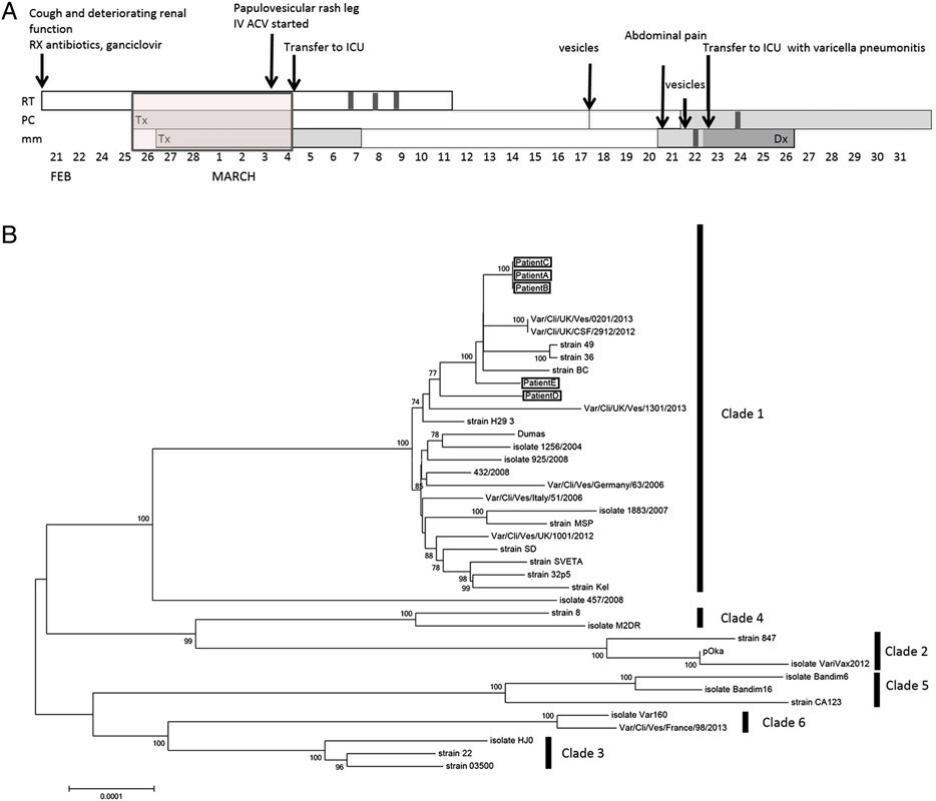
*A*, Timeline showing hospital admissions and clinical histories for patients 1, 2, and 3. *B*, Neighbor-joined phylogeny (100 bootstraps) comprising the 3 linked cases (patients 1, 2, and 3) and 2 unlinked cases (patients 3 and 4), together with 35 varicella-zoster virus database sequences obtained from GenBank. Abbreviations: Dx, diagnosis; ICU, intensive care unit; Pt, patient; Tx, treatment.

A 55-year-old woman with end-stage renal failure caused by renovascular disease received a cadaveric renal transplant (MM110; donor negative/recipient positive for human cytomegalovirus [HCMV]), followed by basiliximab induction and triple therapy with tacrolimus, mycophenolate mofetil, and prednisolone. After receiving the transplant, she was an inpatient in a 4-bed bay for 9 days. Fourteen days after discharge she was readmitted into isolation with abdominal pain, a macular rash, and abnormal liver function tests. The rash vesiculated within 24 hours and a clinical diagnosis of varicella was made. She was treated with intravenous acyclovir (10 mg/kg 3 times daily), antibiotics, and cessation of immunosuppression. Three days after admission she was transferred to the ICU, where she received inotropic and ventilator support. She died 3 days later. Varicella pneumonitis was diagnosed, and it was noted that the patient had been VZV immunoglobulin (Ig) G negative before transplantation and had not received varicella vaccine. She had had no known contact with persons with varicella or zoster (Figure 1*A*; Supplementary Figure 1).

### Patient 2

A 61-year-old male patient with advanced renal failure, due to IgA nephropathy with secondary focal segmental glomerulosclerosis, received a preemptive live donor renal transplant (MM121; donor negative/recipient negative for HCMV). He received basiliximab induction, followed by tacrolimus, mycophenolate mofetil, and prednisolone. Despite a history of chickenpox in childhood he was noted to be IgG VZV negative before transplantation. He remained on the transplant ward for 7 days. Eighteen days after discharge, he was readmitted to a different ward with a 4-day history of thoracic vesicular rash, fever, and abnormal liver function. Disseminated shingles was diagnosed, and the presence of VZV DNA was confirmed by polymerase chain reaction. The patient was immediately isolated and treated with intravenous acyclovir (10 mg/kg 3 times daily). He completed 7 days of intravenous acyclovir, followed by 1 week of oral acyclovir. Five weeks after the original presentation, he had further reactivation of VZV in the L1 dermatome and received 2 further weeks of oral acyclovir and 3 months of prophylactic treatment. He made a full recovery and retained good graft function (Figure 1*A*; Supplementary Figure 1). Importantly, a retrospective review indicated that disseminated shingles was a misdiagnosis and that recurrent varicella should have been diagnosed.

### Patient 3 (Index Case Patient)

A 67-year-old man was admitted into respiratory isolation on the transplant ward, with cough and deteriorating renal function after dual cadaveric renal transplant 4 weeks previously. He was isolated immediately and treated with antibiotics. Low-grade HCMV viremia (3.92 × 10^2^/mL) was treated with valgancyclovir (450 mg/d). On day 10 after admission the patient was noted to have a papulovesicular rash on his right leg. A clinical diagnosis of shingles was confirmed, and treatment was started with intravenous acyclovir (10 mg/kg 3 times daily). Within 24 hours, the rash became generalized, respiratory failure developed, and the patient was transferred to the ICU. He recovered with a fully functioning graft. Because this patient was isolated and did not initially have a rash while patient had received a diagnosis of shingles, nosocomial transmission was not suspected. However, further investigation after the death of patient 1 identified a period when all 3 patients had been inpatients on the same ward, albeit physically separated from patient 3. To investigate whether the 3 cases were linked, we genotyped and sequenced samples from all 3 patients and 2 patients with unrelated cases that occurred in the hospital at the same time (Figure 1*A*, Supplementary Figure 1).

### Genotyping

Residual DNA samples were extracted using the QIAamp DNA Mini Kit (Qiagen) according to the manufacturer's instructions. Amplification and capillary sequencing of a 447–base pair region of open reading frame 22, containing 4 single-nucleotide polymorphisms (SNPs) that discriminate between clades 1/3, 2, 4, and 5 (SNPs 37902, 38055, 38081, and 38177) was carried out as described elsewhere [6]. In addition, the highly variable repeat region, origin of replication (OriS), which also discriminates clade 1 from other clades, was amplified and capillary sequenced using conditions published elsewhere [2].

### Whole-Genome Sequencing and Assembly of VZV

Sequencing libraries were constructed as described elsewhere [7, 8] and sequenced on an Illumina MiSeq Sequencer. Data sets were trimmed and aligned against VZV strain Dumas (NC_001348.1) using BWA (version 0.7.12) [9] and SAMTools software (version 1.0) [10]. Duplicate reads were removed, and a consensus sequence for each data set generated using in-house PERL scripts. Iterative repeat regions (R1, R2, R3, R4, and R5) and the terminal repeat region were excluded from analyses. OriS sequences were successfully captured within the read data and used to verify polymerase chain reaction–based results for all samples. Consensus sequences were aligned using MAFFT software (version 6) [11], and a neighbor-joined phylogenetic tree, based on the Tamura-Nei model, was inferred using MEGA software (version 6.06) [12]. Genome sequences are available in GenBank (accession Nos. KP771912, KP771912, and 3 more awaiting assignment by GenBank as of 16 May 2016).

## RESULTS

Virus from all 3 renal patients and 2 “control” patients were typed as clade 1 by SNP genotyping, and all 5 had identical OriS sequences. Whole-genome sequencing showed the 2 putatively linked patients to be 100% identical to the index case patient, patient 3 (Figure 1*B*) but not the control case patients, whose sequences contained 21 (patient 4) and 11 (patient 5) SNP differences across the genome. Phylogenetic analysis confirmed the clustering of the linked cases, whereas the 2 unlinked clade 1 viruses recovered from other patients in the hospital clustered separately from the linked cases and from each other (Figure 1*B*).

## DISCUSSION

We report the use of whole viral genome sequencing to confirm an unusual case of nosocomial VZV transmission that occurred despite source isolation of the index case patient. Although the 3 patients were in adjacent rooms for more than a week, no immediate connection between them was suspected, partly because shingles was not diagnosed in the index case patient until just before his transfer to the ICU and partly because there was no direct contact between patients. Conventional genotyping confirmed all 3 patients to be infected with clade 1 viruses, the most common genotype in European populations [7]. However, SNP genotyping failed to differentiate the putatively linked viruses from 2 epidemiologically unrelated viruses sampled from other patients at about the same time. Sequencing of highly variable regions, such as the R1 tandem repeat located in open reading frame 11 and the OriS, has also been used to investigate nosocomial transmission [2, 8]. In the cases we report, both linked case patients and controls had identical OriS sequences, and identical R1 sequences in unrelated viruses have also been observed [2, 8].

Although amplicon sequencing to track nosocomial and other transmission events is well established for small RNA viruses including human immunodeficiency virus and hepatitis C virus [9, 13], whole-genome sequencing is required for larger DNA viruses, because these are genetically less variable. To this end, we have developed a method to sequence whole pathogen genomes directly from clinical material [10]. Our data show, unequivocally, the identity between the viruses recovered from the index case patient and 2 contact case patients. The putatively linked viruses were phylogenetically distinct from other clade 1, viruses including 1 recovered contemporaneously in the same hospital.

Although airborne and environmental transmission of virus from cases of zoster to distant contacts have previously been reported [4], it has not hitherto been possible to confirm nosocomial spread, other than in rare cases where a unique signature sequence was present in an amplicon used for SNP typing [4]. Airborne transmission despite source isolation has been described previously, particularly where viral loads in the index case patient are high (eg, in immunosuppressed patients and those with hemorrhagic varicella). Thus, the need for strict respiratory isolation of such patients, preferably in negative pressure rooms, is paramount [4].

Our case report also underlines 2 important clinical issues. First, primary varicella can be fatal in immunosuppressed patients despite appropriate early treatment and support. VZV antibody testing and immunization against VZV in patients for whom renal transplantation is planned has been shown to prevent or attenuate varicella [14]. Second, widespread rash in an immunosuppressed patient with a history of chickenpox, as in our patient 2, cannot be assumed to be disseminated shingles. Identifying cases of recurrent chickenpox due to nosocomial transmission is important for assuring infection control measures. This case report illustrates how whole-genome sequencing can contribute to this process.

In summary, whole VZV genome sequencing directly from clinical material provides a tractable method for monitoring nosocomial transmission. Applying automated methods directly to residual diagnostic samples, we can generate results within 5 days of sample receipt. The data provide support for the risk of airborne transmission of VZV, especially when the viral load is high, and underline the vulnerability of immunocompromised patients to serious infection, irrespective of varicella history. Our report highlights the ongoing need for vaccines that can be prevent viral reactivation in immunocompromised patients as an important measure for control to prevent nosocomial spread.

## Supplementary Data

Supplementary materials are available at http://jid.oxfordjournals.org. Consisting of data provided by the author to benefit the reader, the posted materials are not copyedited and are the sole responsibility of the author, so questions or comments should be addressed to the author.

Supplementary Data
