# Supplementary material for: Viral Genome Sequencing Proves Nosocomial Transmission of Fatal Varicella
Source: J Infect Dis. 2016 Aug 28;214(9):1399–402. doi: 10.1093/infdis/jiw398 (PMC5079377; doi:10.1093/infdis/jiw398)
Supplement: Supplementary Data [file supp_214_9_1399__index.html]

Viral genome sequencing proves nosocomial transmission of fatal varicella — Viral Genome Sequencing Proves Nosocomial Transmission of Fatal Varicella — Supplementary Data 

# Viral Genome Sequencing Proves Nosocomial Transmission of Fatal Varicella

## Supplementary Data

Supplementary Data

- Supplementary Figure 1 - docx file
