# Supplementary material for: Viral Genome Sequencing Proves Nosocomial Transmission of Fatal Varicella
Source: J Infect Dis. 2016 Aug 28;214(9):1399–402. doi: 10.1093/infdis/jiw398 (PMC5079377; doi:10.1093/infdis/jiw398)
Supplement: Supplementary Data [file supp_jiw398_jiw398supp_fig1.docx]

Supplementary Figure S1: Schematic of transplant ward indicating location of patients referred to in this study.
